# A scoping review of obesity education interventions for current and prospective medical professionals in Canada

**DOI:** 10.1016/j.obpill.2023.100085

**Published:** 2023-08-19

**Authors:** Taniya S. Nagpal, Nicole Pearce, Sanjeev Sockalingam, Raed Hawa, Khushmol K. Dhaliwal, Dayna Lee-Baggley, Mohamed El-Hussein, Sarah Nutter, Helena Piccinini-Vallis, Michael Vallis, Liz Dennett, Mary Forhan, Stasia Hadjiyanakkis, Robert F. Kushner, Michelle McMillan, Sean Wharton, David Wiljer, Joseph Roshan Abraham

**Affiliations:** a Faculty of Kinesiology, Sport, and Recreation, University of Alberta, Edmonton, Alberta, Canada; b Obesity Canada, Edmonton, Alberta, Canada; c Department of Psychiatry, University of Toronto, Centre for Addiction and Mental Health, Toronto, Ontario, Canada; d Department of Psychiatry, University of Toronto, Toronto, Ontario, Canada; e Faculty of Medicine and Dentistry, University of Alberta, Edmonton, Alberta, Canada; f Department of Family Medicine, Dalhousie University, Halifax, Nova Scotia, Canada; g Faculty of Health, Community & Education, School of Nursing and Midwifery, Mount Royal University, Calgary, Alberta, Canada; h Educational Psychology and Leadership Studies, University of Victoria, Victoria, British Columbia, Canada; i Scott Health Sciences Library, University of Alberta, Edmonton Alberta Canada; j Department of Occupational Science and Occupational Therapy, Temerty Faculty of Medicine, University of Toronto, Toronto, Ontario, Canada; k Children's Hospital of Eastern Ontario, University of Ottawa, Ottawa, Ontario, Canada; l Northwestern University Feinberg School of Medicine, Chicago, IL, USA; m University of Toronto, Wharton Medical Clinic, Toronto, Ontario, Canada; n University Health Network; Department of Psychiatry, University of Toronto, Toronto, Ontario, Canada

**Keywords:** Obesity, Education, Medicine, Canada

## Abstract

**Background:**

Obesity is a prevalent chronic disease in Canada. Individuals living with obesity frequently interact with medical professionals who must be prepared to provide evidence-based and person-centred care options. The purpose of this scoping review was to summarize existing educational interventions on obesity in Canada for current and prospective medical professionals and to identify key future directions for practice and research.

**Methods:**

A scoping review was conducted following the Preferred Reporting Items for Systematic Reviews and Meta-Analyses extension for scoping reviews. The search strategy was conducted using Medline (via PubMed), Embase, Eric, CBCA, Proquest Education, and Proquest Theses. The inclusion criteria included delivery of an educational intervention on obesity for current medical professionals, medical undergraduate trainees, or residents administered in Canada. Data were extracted from the included studies to thematically summarize the intervention content, and main outcomes assessed. Future directions for research and practice were identified.

**Results:**

Eight studies met the inclusion criteria. The interventions ranged in terms of the mode of delivery, including interactive in-person workshops and seminars, online learning modules, webinars, and videos. The main outcomes assessed were attitudes towards patients living with obesity, self-efficacy for having sensitive obesity-related discussions, skills to assess obesity and provision of management options. All studies reported improvements in the outcomes. Future directions identified were the need to develop standardized obesity competencies for inclusion across medical education programs, further research on effective pedagogical approaches to integrating content into existing curricula and the need for broader awareness and assessment of the quality of obesity education resources.

**Conclusion:**

Although there have been few obesity-specific educational interventions for current and prospective medical professionals in Canada, existing evidence shows positive learning outcomes. These findings advocate for continued investment in the development of obesity medical training and educational interventions.

## 1 Introduction

Obesity is a complex chronic disease and is widely recognized as a global health concern that requires multifaceted and person-centred management options [[1], [2], [3], [4], [5]]. The most recent and updated definition of obesity, that extends beyond using body mass index (BMI) as a diagnostic criterion, characterizes the condition by dysfunctional or excessive adipose tissue that impairs health [1]. Persistent calls to action have been made to implement effective strategies to prevent and manage obesity [2,4]. Most recently, the Canadian Adult Obesity Clinical Practice Guidelines were published and cited as the first wide-ranging resource on obesity care that acknowledge its multifactorial etiology, progressive nature, and need for comprehensive management options that could be inclusive of medication, psychological and behavioural therapy, surgery, lifestyle behaviours and social support [1,6]. To ensure that the most recent, relevant, and evidence-based guidelines are effectively implemented, it is imperative that educational efforts for current and prospective healthcare professionals are aptly updated.

Healthcare professionals play a vital role in obesity management [7]. Patients with obesity will likely interact with several healthcare professionals from a variety of disciplines, in addition to their primary care providers [8]. Unfortunately, studies that have assessed healthcare provider confidence and knowledge about delivering care to patients with obesity show disappointing results such as low self-efficacy to discuss obesity, lack of awareness of referrals for behavioural and psychological therapy, medication or surgery, and biased attitudes towards health behaviours predominantly assuming that their patients are inactive or have poor dietary habits [[9], [10], [11], [12]]. In fact, a recent survey of 591 primary care providers in Canada found that more than 50% did not feel comfortable referring their patient to medical or surgical options of care for obesity, and only 25% felt prepared to follow up with a patient who has undergone bariatric surgery [10]. Similarly, a survey administered to Canadian undergraduate medical students noted that most felt they are not ready to have weight or obesity management related discussions with their future patients [13]. It is apparent that educational initiatives are urgently needed to equip healthcare professionals with the knowledge, confidence, and resources to offer high-quality patient-centred care for obesity.

Previous studies that have tested educational interventions on obesity in medical education have shown promising results for improving overall knowledge on obesity pathophysiology and self-efficacy for engaging in weight counselling and providing referrals to allied health professionals [[14], [15], [16], [17]]. For example, Velazquez et al. implemented a 2.5-day obesity workshop for medical residents and fellows from various medical disciplines in the United States [14]. The workshop included sessions on the causes of obesity, weight counselling, and treatment approaches, spanning from medical intervention to behavioural support [14]. Pre-and post-intervention levels of knowledge and competence on obesity were analysed through survey measures and all participants reported improvement in their understanding of obesity, and at a 6-month follow up, indicated that participating in the workshop positively affected their confidence when caring for patients who have obesity in clinic [14]. Similar results were reported by Koran-Scholl et al., who implemented an intervention inclusive of a 15-min ​educational video on patient-centred care for family doctors in the United States [15]. Participants reported that watching the video increased their comfort in discussing obesity treatment options with their patients [15].

To effectively manage obesity, it is essential that medical education provides prospective and current professionals with up-to-date knowledge of guidelines and directives for patient care [18]. In fact, international consensus statements on obesity care and prevention of weight bias emphasize that healthcare professionals are at the front-line and educating them is of critical importance for a population-level approach to obesity management [2,3,19]. Equipping healthcare professionals with appropriate knowledge, skills, attitudes and resources on obesity management will result in the translation of this knowledge to patient care, and accordingly, have a downstream effect on overall health for individuals living with obesity [19].

In Canada, obesity was recognized as a chronic disease by the Canadian Medical Association in 2015 and the first comprehensive clinical practice guideline on obesity management was released in 2020 [1]. Moving forward, it is necessary to integrate and standardize effective educational interventions in the Canadian medical education curriculum on obesity management in line with recent guidelines. Accordingly, this scoping review aimed to identify and describe Canadian medical education interventions for obesity that have been developed and tested. A scoping review aims to map the literature on a specific topic, with the goal of identifying similarities or common themes to summarize findings [20]. A scoping review may precede other types of reviews, such as a systematic review, as they are meant to be a broader overview and address an emerging or evolving topic [20,21]. In this scoping review, the data were categorized to summarize the mode of delivery, content, outcomes assessed, and results of the educational intervention. These findings will help inform the development of Canadian medical education competencies on obesity management and offer suggestions on educational methods that could be incorporated into existing curricula.

## 2 Methods

This scoping review was completed in accordance with the 2020 PRISMA-ScR extension for scoping reviews [20]; checklist is included as an Appendix. The main objective of this review was to identify and describe educational interventions on obesity that have been developed and tested within Canadian medical education curricula or as a continuing education opportunity for medical professionals.

### 2.1 Search strategy

A health sciences librarian developed and implemented the search strategy. The search strategy included 3 main concepts: obesity, Canada, and education; the full search strategy for Medline is presented in Table 1. The following databases were searched: Medline (via PubMed), Embase (Ovid interface), Eric (Proquest interface), Education Database (Proquest interface), Canadian Business & Current Affairs Database (Proquest interface), and Theses and Dissertations Global (Proquest interface). The search strategy was optimized for each database. This search was carried out on February 13, 2023, with no date or language restrictions in place; however only English language articles were extracted.

**Table 1:** Search strategy for medline.

1 exp ∗Education/ or ∗students/ or exp ∗students, health occupations/ 622871 2 (student∗ or teach∗ or educat∗ or (resident not (tissue-resident or resident-macrophage∗)) or residency or PGME or UGME or UME or curricul∗ or school∗ or university or college or undergraduate or graduate or post-secondary or professional development or pedagog∗ or module∗ or taught or unit or course or courses).ti,kf. 810569 3 1 or 2 1110505 4 obes∗.mp. 429890 5 exp Canada/ 179487 6 (Whitehorse or Yellowknife or Iqaluit or ((Vancouver or Victoria or Burnaby or Surrey or Langley or Kelowna or Kamloops or “Prince George” or Richmond or Abbotsford or Nanaimo or Kwantlen or Capilano) adj2 (BC or CAN)) or ((Edmonton or Calgary or Athabasca or Lethbridge or “Medicine Hat” or Lacombe) adj2 (AB or ALTA or CAN)) or ((Saskatoon or Regina) adj (SK or Sask or CAN)) or ((Winnipeg or Brandon or “The Pas”) adj2 (MB or MAN or CAN)) or ((Ottawa or Toronto or North Bay or Thunder Bay or Sudbury or “Sault Ste. Marie” or Peterborough or Kingston or London or Hamilton or Kitchener or Windsor or “St. Catharine∗" or Guelph or Waterloo or (York not New York)) adj2 (ON or ONT or CAN)) or ((Montreal or Concordia or Gatineau or “Trois Rivieres” or Sherbrooke or Chicoutimi) adj2 (QC or QUE or PQ or CAN)) or ((Fredericton or Moncton or “Saint John” or Sackville) adj2 (NB or CAN)) or (Charlottetown adj PEI) or ((“St. John's" or “Saint John's") adj2 (NL or NFLD or CAN)) or ((Acadia or Halifax or “Cape Breton” or Dalhousie or Antigonish or Sydney or Wolfville) adj2 (NS or CAN))).mp,in. 292439 7 (Acadia University or Algoma University or Ambrose University or Aurora College or Bishop∗ University or Brock University or Cape Breton University or Capilano University or Carleton University or College of the North Atlantic or College Universitaire Dominicain or (Concordia University adj6 (Montreal or Edmonton or AN or PQ or QC or QUE or CAN)) or Crandall University or Dalhousie University or Ecole de Technologie Superieure or Ecole Nationale d'Administration Publique or Ecole Polytechnique de Montreal or HEC Montreal or Kingswood University or Kwantlen Polytechnic University or Lakehead University or Laurentian University or MacEwan University or McGill University or Universite McGill or McMaster University or (Memorial University adj4 (NL or nfld or Saint John∗ or “st. John∗")) or Mount Allison University or Mount Royal University or Mount Saint Vincent University or Nicola Valley Institute of Technology or Nipissing University or Northern Alberta Institute of Technology or NSCAD University or OCAD University or Ontario Tech University or (“Queen's University” adj6 (ON or ONT or Kingston or CAN)) or Royal Roads University or Ryerson University or (“Saint Mary's University” not “Saint Mary's University of Minnesota”) or SAIT Polytechnic or Simon Fraser University or “St. Francis Xavier University” or “King's University” or University of Winnipeg or Thompson Rivers University or (Trent University not Nottingham Trent University) or Trinity Western University or Universite de Hearst or Universite de Moncton or University of Moncton or Universite de Montreal or University of Montreal or Universite de Saint-Boniface or Saint-Boniface University or Universite de Sherbrooke or University of Sherbrooke or Universite Laval or Laval University or Universite Sainte-Anne or University of Alberta or University of Calgary or University of Guelph or University of Lethbridge or University of Ottawa or University of Regina or University of the Fraser Valley or University of Toronto or University of Waterloo or University of Windsor or Vancouver Island University or (Western University adj6 (London or on or ONT or CAN)) or Wilfrid Laurier University or (York University not New York University) or Yorkville University).mp,in. 643328 8 exp africa/ or exp caribbean region/ or exp central america/ or latin america/ or greenland/ or mexico/ or exp united states/ or exp south america/ or antarctic regions/ or arctic regions/ or exp asia/ or exp europe/ or exp oceania/ 4570609 9 3 and 4 and ((5 or 6 or 7) not (8 not 5)) 660

### 2.2 Inclusion and exclusion criteria

For inclusion in this review, the following criteria were met: 1. Inclusion of any kind of educational intervention on obesity; 2. The educational intervention was delivered or integrated within undergraduate medical education, postgraduate medical education or as a continuing education opportunity for practicing medical doctors; 3. The educational interventions were conducted in Canada. The focus of this review was specifically Canada as findings are to be used to inform national directives for obesity medical education, advocacy, and research. Only primary data sources were accepted, including randomized controlled trials, cohort studies, cross-sectional studies, and qualitative investigations. Editorials, commentaries, reviews, guidelines, protocols, opinion papers, and animal studies were excluded. In addition, no articles were included where the purpose of the study was to assess perspectives or knowledge on obesity without the delivery of an educational intervention or program.

### 2.3 Screening process

All retrieved articles were transferred to the Covidence software and duplicate articles were removed. Each article was reviewed by two independent reviewers first by assessing the titles and abstracts for inclusion. Following this, all articles were independently reviewed by two reviewers again in their full text for further assessment. If there was a disagreement at any stage, a third reviewer was consulted to achieve consensus.

### 2.4 Data extraction

The included studies underwent data extraction using a standardized excel spreadsheet. The following data were extracted to describe the educational interventions: mode of delivery of intervention, topics addressed, length of intervention, main outcomes and results, who delivered the intervention, and the audience including sample size and characteristics (e.g., type of medical professional, and year of medical training).

### 2.5 Data mapping

Descriptions of the medical education interventions were synthesized narratively, and an overall summary was provided on the types of interventions administered, the audience, content, and outcomes. Next, the research team categorized the extracted data into themes to identify key directives for future educational initiatives on obesity management.

## 3 Results

**Fig. 1:**
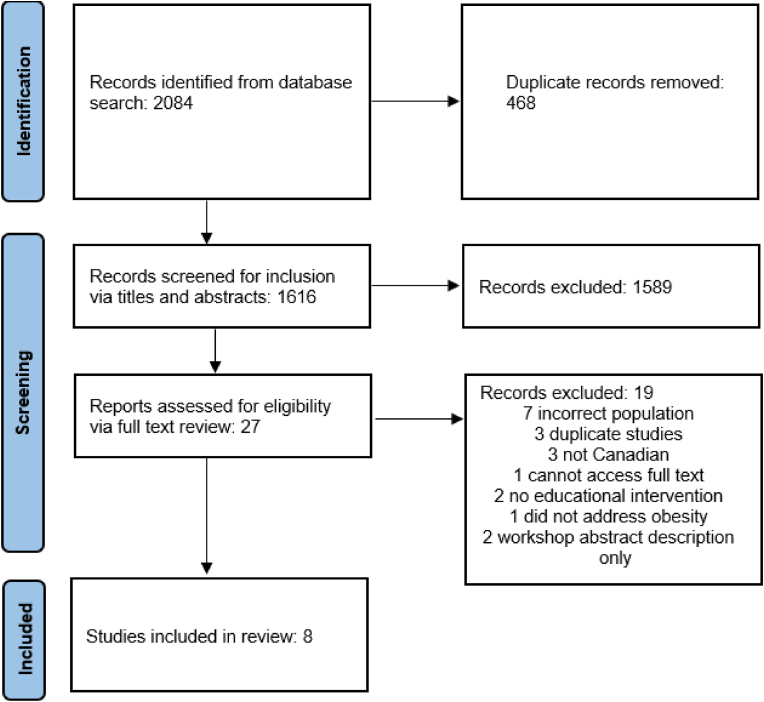
PRISMA flow diagram.

The initial search yielded 2084 articles, and after removal of duplicates, 1616 articles underwent screening. Eight studies met the inclusion criteria (n = 8; Fig. 1 depicts the screening process). Of note, two excluded abstracts were identified that either described a workshop or detailed an upcoming workshop, however information about content or results were not available online [22,23]. Among the eight studies included in this scoping review, four had in-person interactive sessions or workshops [[24], [25], [26], [27]], four offered material online [24,25,28,29], one evaluated a training program specifically for performing laparoscopic sleeve gastrectomy [30], five used cased studies delivered through various modalities such as videos and vignettes [24,25,[27], [28], [29]], and one focused on providing skills for motivational interviewing [31]. Additionally, four studies integrated patient chart reviews of the trainee's actual patients to assess if there were any changes in their delivery of care following the educational intervention [24,[29], [30], [31]]. Main outcomes assessed were changes in provider attitudes towards their patients or prospective patients who have obesity (n = 4); [[24], [25], [26], [27]], knowledge on obesity and obesity management (n = 7); [24,25,[27], [28], [29], [30], [31]], and self-efficacy for discussing obesity and providing care (n = 6); [24,25,27,[29], [30], [31]].

All of the included studies reported improvements in outcomes following the educational intervention. Table 2 provides a detailed overview of each intervention, outcomes assessed, and the audience. Based on these summaries, the following critical gaps in medical education on obesity in Canada were identified.

1. Despite the favourable results, the majority of interventions included a small sample size and may be subjected to selection bias, thus pointing to the need to implement standardized obesity competencies in curricula to reach a wider and consistent audience;
2. The method of content delivery varied across studies and therefore it is necessary to determine effective and feasible strategies to recommend for integration into medical curricula;
3. Overall there is a paucity of published literature on medical educational interventions on obesity in Canada, especially after the release of the 2020 guideline. This is a critical area for future research and advocacy.

**Table 2:** Description of included studies.

Author, Year	Intervention Description	Main Outcomes Assessed and Results	Audience Description
Baillargeon, 2020 [23]	● A 2-day obesity preceptorship was offered. ● Interactive sessions were delivered by content experts including lessons on obesity evaluation, medical and surgical treatment options, behavioural approaches, tips for diet and physical activity, and observation of patient encounters. ● Day 2 of the preceptorship occurred 1 month later. The focus of day 2 was pediatric obesity, nutrition and physical activity, and discussion of clinical vignettes. ● All participants had access to a website with resources and literature on obesity management. ● After the preceptorship there were optional monthly meetings to continue to network with the tertiary team and attend educational webinars.	● Main outcomes were attitudes towards patients who have obesity, confidence for obesity management, self-efficacy for discussing nutrition and physical activity, and a chart review was completed to assess changes in variables that were recorded. ● Physician attitudes towards patients with obesity significantly improved, there was no difference in attitudes of nurses. ● Confidence levels for obesity management significantly improved for all participants and this was maintained at 1 year post. ● Self-efficacy for discussing nutrition and physical activity improved for all participants and this was maintained at 1 year post. ● Physicians were more likely to measure weight, waist circumference, and readiness for change after the training.	35 participants including 12 nurses and 23 physicians.
Garneau, 2014 [29]	● A laparoscopic sleeve gastrectomy preceptorship and proctorship was offered. ● Training was delivered by specialists in bariatric surgery. ● First trainees were brought to a bariatric centre to learn through observation and assist in bariatric procedures. ● Then onsite consulting surgeons offered continuing training. Support personnel such as nurses also attended trainings.	● Outcomes assessed were preoperative, intraoperative and postoperative data of patients. ● The trained surgeons performed appropriately with minor complications on 31 patients assessed.	2 surgeons participated in the training.
Hawa, 2017 [27]	● A resource that provides a case study of binge eating disorder was made available. ● The resource includes video clips to demonstrate the evolution of the case over 10–12 weeks, and has interactive quizzes, texts and other supplementary videos. ● The module explains why medical students need to understand binge eating disorders and how it affects individuals including those who have obesity. ● They review pathophysiology, comorbidities like obesity, and treatment options.	● Feedback on the module has been positive from both students and faculty. ● Comments received included positive feedback on the practicality of the resource overall, improvement in understanding of binge eating disorders and the relationship with obesity, and ease of use.	5 medical students provided feedback.
Luig, 2020 [24]	● The course focuses on the 5 As approach to obesity management. ● The course is available online and is completed in 8–11 h over 2 days. ● The 5As tool was reviewed in interactive lectures and practiced with patients. ● Participants could also wear an empathy suit and engage in tasks of daily living and then complete a one-page narrative reflection. ● Participants engaged in patient interviews using the 5As and then had small group discussions. ● They then were asked to practice their learned skills in clinic and reflect on their experience in a one-page narrative. ● The course is delivered by medical professionals with content expertise.	● Main outcomes assessed were attitudes towards patients who have obesity, confidence in providing care, and changes in practice. ● Overall, following the course beliefs about obesity improved however there was no change in attitude. ● Residents were more confident in caring for patients who have obesity including assessing causes, advising on treatment options, weight counselling, and providing referrals.	42 family medicine residents.
Obadia, 2013 [30]	● A motivational interviewing training intervention was delivered to family physicians on childhood obesity management. ● This was compared with receiving standard obesity-related educational resources. ● Patient charts were reviewed of physicians who attended the training to compare differences in recorded care.	● 246 patient charts were reviewed. ● The motivational interviewing group was more likely to record pre-existing medical conditions and provide a referral to an allied health professional.	12 primary care physicians completed the trainings.
Ryan, 2017 [25]	● An evidence-based physical activity education intervention was developed by content experts. ● This was administered via a seminar focused on benefits of being active, and how to integrate physical activity in clinical settings. ● Health communication strategies were discussed to empathetically engage in physical activity counselling with individuals who have obesity. ● Weight bias content was also included.	● Beliefs and attitudes about persons who have obesity were measured pre and post the seminar and overall there was a statistical improvement.	Majority of the participants were in undergraduate health-related programs, with only 1 who specified they are currently training in medicine.
Sanchez-Ramirez, 2018 [26]	● A one-day accredited educational event was organized by a team of allied health professionals. ● The goal was to promote interprofessional learning to care for people who have obesity. ● Leaders presented on their experiences caring for patients who have obesity. ● Obesity case studies were reviewed followed by round table discussions. ● Topics covered were the role of healthcare professionals in obesity management, prevalence, prevention, collaborative approaches to care, barriers in discussing weight loss, effective counselling, assessing readiness for change and opportunities to practice having patient conversations.	● Main outcomes assessed were skills, attitudes and challenges towards obesity management. ● Overall there was improvement in skills to assess weight and address weight management issues. ● Participants felt more knowledgeable about obesity, were more aware of where to refer patients and comfortable having discussions on obesity. ● These improvements remained after 6 months.	Front-line healthcare providers were invited to attend including physicians; 67 completed both pre and post evaluations.
Wharton, 2022 [28]	● An online self-directed platform titled Integrated Approaches to Change Treatment in Obesity (i-ACT™ in Obesity). ● The goal of the program is to evoke behaviour change and advance the clinical management of people with obesity. ● It is developed by medical professionals and experts in the field of obesity management. ● The platform includes self-teaching and interactions with mentors and obesity experts. ● Content includes understanding the learner's profile to individualize the curriculum for their needs and knowledge. ● There are educational videos on a variety of topics including consequences of obesity, nutrition, exercise. ● Learners identified 10 patients in their practice to reflect on how their practice has changed. ● There was opportunity to receive feedback in accordance with Obesity Medical Education Collaborative competencies. ● Small virtual groups sessions were available for mentoring.	● Overall pilot results showed that there was significant improvement in confidence and comfort to have obesity related discussions with patients and greater intention to use more obesity treatment options moving forward. ● Participants also indicated high satisfaction with using a self-directed learning tool.	91 family physicians participated.

### 3.1 Absence of standardized inclusion of obesity in medical education

The eight included interventions in this review were either an optional course offering that students may decide to partake in, a workshop a trainee may choose to attend, or they were integrated into a residency or preceptorship program for a specific speciality. Accordingly, this means that the included interventions may be subjected to selection bias as perhaps the learners who participated had a vested interest in obesity medicine. In addition, overall sample sizes ranged from one to 91 medical professionals or trainees, and it was noted in some studies that completion rates of the training and submission of follow up assessments were low [24,26,27]. Of note, Ryan et al. had a total sample size of 24 participants who were undergraduate or graduate trainees in health programs, however only one participant specified they were currently studying medicine [26]. The Obesity Medicine Education Collaborative (OMEC) developed obesity medical education competencies for undergraduate, graduate and fellowship programs and one included study incorporated these into their learning platform [29]. Wharton et al. designed the Integrated Approaches to Change Treatment in Obesity (i-ACT™ in Obesity) platform which is based on the OMEC competencies [29]. However, this platform along with inclusion of the OMEC competencies are not mandatory across medical programs in the country. Given the increasing demand from health professionals themselves for obesity-related training, it would be prudent to integrate such education into existing medical curriculum for all prospective health professionals.

### 3.2 Variability in content delivery

Among the eight interventions, content delivery and what it included varied. There were intensive interventions that included both in-person and online components of content delivery, as well as self-directed learning opportunities. Baillargeon et al., 2020 offered a two-day intensive in-person interactive workshop delivered by content experts [24]. The workshops were comprehensive and covered several topics related to obesity management involving surgical options, medical interventions, and behaviour change [24]. Similarly, Sanchez-Ramirez et al., organized a one-day interactive interprofessional training opportunity which included review of case studies and roundtable discussions [27]. The one-day event addressed topics such as obesity prevention, prevalence, comorbidities, collaborative approaches to care, barriers to weight loss and assessing readiness for change [27]. In both interventions, favourable results were found for improved skills and self-efficacy for obesity management such as feeling confident to have weight-related discussions, measuring obesity and offering patient-centred care [23,26]. Other studies offered the majority of their resources online including video clips of patient cases, webinars, and interactive lectures [25,28,29]. In these studies, positive results were also reported such as improved attitudes towards patients who have obesity, overall knowledge on obesity and increased likelihood to refer care to allied health professionals.

Included content was also variable across the eight studies. Two studies were very specific based on the professional group that was being addressed, including surgeons receiving training for laparoscopic sleeve gastrectomy and addressing binge eating disorders with medical students interested in psychology [28,30]. Ryan et al. primarily focused on education related to physical activity in clinical settings in their seminar, however they also included lessons on health communication to reduce weight bias [26]. Obadia et al. provided training on motivational interviewing for behaviour change [31]. The remaining interventions were more intensive and appeared to address multiple topics over a period of time; consistent topics reported were medical and surgical treatment options for obesity, communication tools with a focus on reducing weight bias, behavioural approaches inclusive of primarily nutrition and physical activity, and pathophysiology of obesity [24,25,27,29].

### 3.3 Paucity of published literature on medical education interventions on obesity in Canada

Irrespective of the positive results, only eight studies were identified that met our inclusion criteria with a total of 243 medical professionals or professionals in training included. It should be acknowledged though, that there are likely several training platforms and resources that are readily available on obesity management that have not been evaluated for their effectiveness and/or findings have not been published. Accordingly, a future research direction may be an environmental scan and review of available resources for obesity medical education more broadly in Canada.

## 4 Discussion

This scoping review summarized current, published medical education interventions on obesity in Canada. Eight studies were identified that addressed a variety of topics related to obesity management, with the most common being strategies to discuss obesity, attitudes towards patients who have obesity, and reviewing care options inclusive of medical and psychological/behavioural interventions. The interventions varied in how they were delivered, inclusive of in person workshops and seminars, online resources, and self-directed learning opportunities. Lessons learned and future directives were the need for standardizing obesity education in Canadian medical curricula to reach a larger audience of healthcare professionals, development and testing of pedagogies that would be most effective to integrate into existing curricula, and broad evaluation of available obesity educational resources through an environmental scan given the lack of researched and published interventions.

Public health messaging on obesity has been criticized for simplifying the narrative on pathophysiology to primarily being an equation of energy balance that is positioned as entirely controlled by the individual's choice to engage or not engage in physical activity and healthy eating behaviours [19]. This basic “eat less, move more” narrative ignores the abundance of evidence supporting the complex etiology of obesity that is caused by an interplay of several physical, environmental, and social factors [32]. Unfortunately, these biased views also are persistent in clinical settings and patients have reported receiving unhelpful and judgemental advice for obesity management from their care providers [[33], [34], [35]]. Experiencing weight bias in a clinical setting is detrimental to patient-provider relationships, resulting in avoidance of care, reduced adherence to treatment regimes, and poor or worsening of mental health outcomes [34]. A direction forward to improve the quality of obesity care, inclusive of reducing biased and simplified views, is to educate medical professionals on appropriate and comprehensive obesity etiology, measurement, and management [19,36]. Medical professionals are often a first point of contact for individuals living with obesity to learn they may have a chronic disease, its progression, and its complex causal pathways and management options [8]. Therefore, it is necessary that medical professionals themselves have appropriate training on obesity management. Positively, recent medical education interventions administered in Canada identified in this review included content on sensitive and inclusive communication strategies, as well as improving provider attitudes [[24], [25], [26], [27],^29^]. Receiving comprehensive obesity education was associated with a reduction in biased attitudes towards patients who have obesity and overall improvements in confidence for having sensitive dialogues with patients [24,25]. The standardization of obesity competencies within the medical curriculum, may increase the likelihood of all medical professionals receiving training that focuses on patient-centred approaches to obesity management and mitigate weight bias in clinical settings.

Two systematic reviews assessed medical education interventions on obesity from around the world [7,37]. First, Katz et al. identified 17 studies specifically addressing undergraduate medical education programs that were primarily administered in the United States [37]. Similar to our findings, common topics addressed were conversation skills, improving attitudes towards patients who have obesity, and information regarding referrals. In addition, mode of delivery for content also varied such as classroom sessions, online self-learning modules, and simulation [37]. Through narrative synthesis, authors concluded that brief educational interventions were effective in achieving objectives, and this was consistent whether the information was delivered in person or through online modalities like videos [37]. In our review as well, the mode of delivery of content varied by type and length, and across all studies positive learning outcomes were reported. A second systematic review assessed obesity education interventions in undergraduate medical education, residencies and fellowships [7]. Mastrocola et al. identified 27 relevant studies and graded each of them for their effectiveness in achieving learning outcomes [7]. Notably, the only learning outcome that achieved an “A grade” was improving counselling confidence [7]. Among the studies that addressed counselling, mode of delivery also differed including interactive sessions and online learning modules [7]. Taken together, further research is needed to identify the best way educational interventions should be delivered on obesity, but there certainly appears to be options for educators to choose from to integrate content efficiently and effectively. An essential step forward in Canada is to develop obesity specific medical competencies, to ensure that prospective and current medical professionals are appropriately prepared to care for individuals who have obesity. Obesity Canada, a national non-profit organization dedicated towards caring for individuals who have obesity through research, education and advocacy, established an Education Action Team that is developing evidence-based obesity medical competencies [38]. An important next step to ensure current and future obesity educational interventions are comprehensive and meet essential healthcare needs would be to evaluate content against expected medical competencies and ensure they are being appropriately addressed.

Notably though, in our review and previous reviews from other regions, limited medical educational interventions on obesity were identified. The low number of results may be because available obesity educational tools are not necessarily researched and published but do exist, and therefore they would not be identified through traditional review methodologies. For example, platforms like Obesity Canada have a range of educational tools and resources located on their Professional Education pages and videos on social media channels that individuals may self-select to participate in or educators may include in their course content [39]. Accordingly, a future research direction may be to conduct a wide environmental scan of non-discipline specific obesity educational resources and an evaluation of the quality of the content, delivery methods, reach and spread, and effectiveness. Promisingly, the few studies from Canada identified in this review showed that education on obesity for current and prospective medical professionals is well received and improves outcomes related to quality of care (i.e., attitudes, skills and self-efficacy). The findings of this scoping review therefore should propel further advocacy initiatives to increase availability of obesity education for all medical professionals. Of note, Obesity Canada includes evaluations for all their educational initiatives; a future direction is to summarize and present these findings to share widely the effectiveness and areas of improvement of obesity education interventions that are already available. In addition, current and prospective educational interventions should be assessed to identify which obesity specific competencies are being adequately addressed. Furthermore, it should be acknowledged with the increasing development and use of Artificial Intelligence, this may be another technological avenue to acquire obesity-related knowledge as well as a tool to use in obesity medicine [40]. For example, use of Artificial Intelligence could enhance educational interventions, such as offering virtual reality options to stimulate practical learning activities [40]. In practice, Artificial Intelligence may be used by healthcare providers to increase efficiency in appointments, such as writing medical summaries, or use of predictive modeling to discuss prognosis [40]. Given that Artificial Intelligence is emerging in educational environments, another key future research direction may be to evaluate this as a tool for advancing obesity education and medical practice.

Strengths of this review include the application of the PRISMA-ScR to guide procedures, the comprehensive search of published literature inclusive of medical curriculum portals, and the thematic assessment of data to identify future directions in obesity medical education. Limitations include the small number of studies and small sample sizes which reflect the need for further research on obesity medical education in Canada. It is important to acknowledge that existing resources may not be captured through traditional scientific databases and review methods, indicating the possibility of missing relevant information. Future work may broaden the scope to be inclusive of all health professionals. These findings highlight a critical need to further develop and test obesity medical education interventions.

## 5 Conclusion

Limited obesity medical education interventions have been developed and tested in Canada, however the few that have, all shown positive results for improving provider attitudes towards patients who have obesity, self-efficacy for having sensitive and patient-centred discussions, and skills for obesity assessment, referrals, and review of management options. Key future directions include the need to develop and implement standardized obesity competencies to integrate into medical programs to ensure that all prospective providers are given comprehensive, updated, and evidence-based training on obesity management. In addition, future research may include investigating effective pedagogies to integrate obesity education into medical curricula, as well as conducting a wider environmental scan to summarize and evaluate existing obesity education resources. Obesity Canada is actively working to develop Canadian obesity medical education competencies; a key future objective will be to evaluate existing and prospective educational initiatives to ensure they are addressing essential competencies to care for individuals who have obesity.

## Authorship statement

TSN, NP, KKD and JRA conceptualized the review with significant input from all co-authors as they are members of the Obesity Canada Education Committee. LD developed the search strategy and conducted the search. TSN, NP, KKD, SS, RH, DL-B, ME-H, HP-V, MV and RA assisted with data screening, extraction, and analysis. All authors assisted with the development of the themes, including deciding upon the recommended future research directions. 10.13039/501100004413TSN prepared the manuscript with support from 10.13039/100006209NP and JRA. All authors reviewed the final manuscript and provided approval.

## Ethical review

As this was a scoping review, ethical review was not required.

## Funding

This research was funded by Obesity Canada's Fund for Obesity Collaboration and Unified Strategies (FOCUS) initiative in addition to in-kind support from the scientific and professional volunteers engaged in the process.

## Declaration of Artificial Intelligence (AI) and AI-assisted technologies in the writing process

During the preparation of this work the authors did not use AI-assisted technologies.

## Declaration of competing interest

The authors declare the following financial interests/personal relationships which may be considered as potential competing interests: Nicole Pearce is an employee of Obesity Canada. Sanjeev Sockalingam is the Scientific Director of Obesity Canada. Mary Forhan is the past Scientific Director of Obesity Canada. All authors are volunteer members of Obesity Canada's Education Committee. No other conflicts to declare.
